# Transjugular Intrahepatic Portosystemic Shunt for Refractory Ascites in Gaucher Disease

**DOI:** 10.7759/cureus.23941

**Published:** 2022-04-08

**Authors:** Kunal Adhyaru, Sherna Menezes, Pramod K Mistry, Aabha Nagral

**Affiliations:** 1 Department of Gastroenterology and Hepatology, Jaslok Hospital and Research Centre, Mumbai, IND; 2 Department of Internal Medicine, Yale University, New Haven, USA; 3 Department of Gastroenterology and Hepatology, Apollo Hospital, Navi Mumbai, Mumbai, IND

**Keywords:** lysosomal storage diseases, cirrhosis, decompensated liver disease, rare disease, inherited metabolic disease

## Abstract

Gaucher disease is rare, inherited lysosomal storage disorder that leads to the excessive accumulation of certain lipids, especially within the bone marrow, liver, and spleen. We present a case of a 30-year-old man with Gaucher disease who underwent a splenectomy at the age of eight for severe cytopenia. His subsequent history was notable for recurrent avascular osteonecrosis and his liver disease progressed to portal hypertension, variceal bleeding, and refractory ascites. Upon evaluation of his candidacy for liver transplantation, he was sarcopenic, with tense, high serum-ascites albumin gradient (SAAG) ascites and florid venous collaterals on his anterior abdominal wall. His hepatic venous pressure gradient (HVPG) was 22 mmHg. He underwent a transjugular intrahepatic portosystemic shunt (TIPS) procedure, following which his HVPG was reduced to 2 mmHg and striking reversal of ascites as well as improvement of his nutritional state. TIPS was not complicated by hepatic encephalopathy. The successful outcome of TIPS in Gaucher disease with advanced hepatic disease underscores its utility as a bridge to liver transplantation with continuing enzyme replacement therapy.

## Introduction

Gaucher disease is a prototype lysosomal disease due to deficiency of lysosomal glucocerebrosidase and multisystemic accumulation of glucocerebroside-laden macrophages [1]. The liver is always involved and in some splenectomized patients, it can progress to advanced decompensated liver disease [2]. The hepatocytes are spared; therefore, the hepatocellular function is preserved despite florid portal hypertension as illustrated in our patient [2]. Enzyme replacement therapy (ERT) is the mainstay of treatment in Gaucher disease and liver transplantation is performed in adjunct in patients with advanced liver disease or cirrhosis leading to refractory symptoms [3]. Consideration of cellular pathology of liver disease impacts clinical decision-making, as non-transplant options should be considered first before transplantation. Indeed after successful Transjugular Intrahepatic portosystemic shunt (TIPS), it offers a window of opportunity for a trial of enzyme therapy.

## Case presentation

Case history

**Figure 1:**
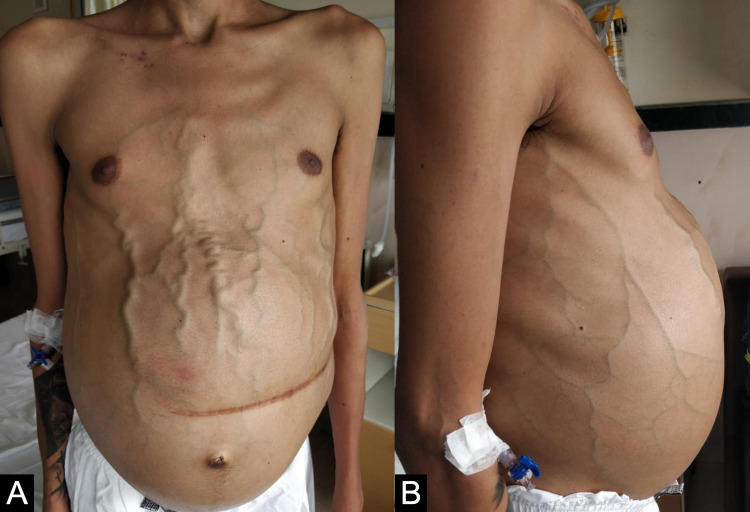
Pre-TIPS procedure. (A) Anterior view of the abdomen showing distended abdomen with a splenectomy scar, markedly dilated veins, gynecomastia, small umbilical hernia and sarcopenia. (B) Lateral view of the abdomen showing dilated veins in the flanks. TIPS - Transjugular Intrahepatic Portosystemic Shunt

We report a case of a 30-year-old man diagnosed at the age of eight with Gaucher disease when he was splenectomized for massive undiagnosed splenomegaly needing repeated blood transfusions. He had Gaucher disease with L444P variation, now renamed Leu483Pro (rs421016). The family was negative on screening. He also had a history of recurrent bone crisis and underwent bilateral hip joint replacements for avascular necrosis of the femoral head at 20 years of age. He had variceal bleeding at the age of 28 years followed by the development of ascites which had become refractory for the past six months. He developed dyselectrolytemia with increasing doses of diuretics and needed frequent paracentesis. He was referred to us for liver transplantation. On examination, he was sarcopenic, had tense ascites and hugely dilated veins on the abdominal wall (Figures 1A, 1B).

Investigations

Investigations revealed deranged liver function tests (LFTs) with elevated bilirubin and transaminases, and decreased albumin levels. His iron profile study was normal and the highly elevated unconjugated bilirubin could be due to an exaggeration of underlying Gilbert's syndrome or hemolysis. International normalized ratio (INR) was elevated. His MELD 3.0 score was 27 and Child-Turcotte-Pugh score was 11. Hematology panel showed mildly reduced hemoglobin with no abnormalities in the other parameters. Beta-glucosidase level was reduced on the lysosomal enzyme study of leukocytes (Table 1).

**Table 1:** Laboratory parameters Abnormal LFTs showing elevated bilirubin and transaminases with hypoalbuminemia noted. Reduced beta-glucosidase levels on enzyme assay is present as seen in Gaucher disease.

Sr. No.	Investigation	Value on admission	Reference Range
1	Hemoglobin	11.2 g/dL	12.0-15.5 g/dL
2	WBC	6.65 (10^3/µL)	3.9-11.7 (10^3/µL)
3	Platelets	263 (10^3/µL)	140-440 (10^3/µL)
4	International Normalized Ratio (INR)	1.46	0.8-1.2
5	Total bilirubin	20.9 mg/dL	0.20-1.30 mg/dL
6	Direct bilirubin	1.2 mg/dL	0.10-0.50 mg/dL
7	Alanine Transaminase (ALT)	59.8 U/L	10-33 U/L
8	Aspartate Transaminase (AST)	114.8 U/L	10-32 U/L
9	Total Protein	8.2 g/dL	6.6-8.7 g/dL
10	Albumin	1.8 g/dL	3.97-4.94 g/dL
11	Beta-glucosidase level	3.44 nmol/hr/mg protein	5.1-11.32 nmol/hr/mg protein

He had a high serum ascites albumin gradient (SAAG) ascites. 2D echocardiogram showed good systolic function with an ejection fraction of 55%. He had not undergone a liver biopsy. His transjugular study revealed a hepatic venous pressure gradient (HVPG) of 22 mmHg (normal - 1 to 5 mmHg). In the genetic mutation study, he was found to have a pathogenic variation in the GBA gene c.1448T>C (rs421016) causing amino acid change of L444P in the beta-glucosidase protein. The pathogenicity was classified as per American College of Medical Genetics and Genomics (ACMG) criteria and Sanger sequencing was done.

Treatment and outcome

In view of the frequent need for large volume paracentesis, a self-expanding metallic stent (SEMS) - 10 mm x 80 mm, “EPIC BOSTON” - connecting the left portal vein to the inferior vena cava was placed during the TIPS procedure. He was also subsequently started on ERT of imiglucerase at 60 units/kg infusion once every 15 days.

**Figure 2:**
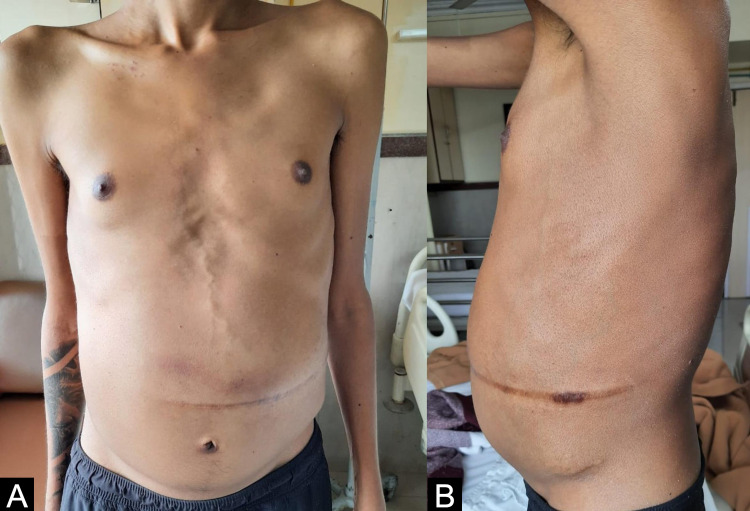
Post-TIPS procedure. (A) Anterior view of the abdomen showing marked reduction in abdominal distension and venous dilation. (B) Lateral view of the abdomen showing marked reduction in abdominal distension and venous dilation. TIPS -Transjugular Intrahepatic Portosystemic Shunt

Post-TIPS procedure, his HVPG fell down to 2 mmHg. The ascites and the dilated abdominal veins resolved significantly after the TIPS procedure over a period of two months (Figures 2A, 2B). He remained well for a few months with mild ascites. However, he underwent paracentesis at an outside hospital when he developed fever which led to hemorrhage and hematoma formation. He presented to us a few days later with increasing abdominal pain and tenderness which was suspected to be due to spontaneous bacterial peritonitis (SBP). He also displayed signs of sepsis and underwent treatment for presumptive SBP, but unfortunately he later succumbed after developing hepatorenal syndrome with septic shock.

## Discussion

Gaucher disease is a rare autosomal recessive disorder that is caused by the deficiency of the enzyme glucocerebrosidase. It results in abnormal accumulation of glucosylceramide in the reticuloendothelial system, including the liver, spleen, and bone marrow [1]. Hepatic manifestations of Gaucher disease are markedly varied. They include hepatomegaly, hepatic fibrosis, cirrhosis as well as hepatocellular carcinoma. Cases with portal hypertension and cholelithiasis have also been reported [2]. There have been various mechanisms described to explain the likely pathophysiology behind liver fibrosis. These include infiltration by Gaucher cells, pro-inflammatory cytokines, splenectomy, and iron overload. The removal of the spleen may result in increased hepatic infiltration leading to further liver injury [4]. In a study of type 1 Gaucher disease patients, significant liver fibrosis was seen in 19% of patients (seven out of 37). It also showed that splenectomy and the severity of the disease are strong predictors of liver fibrosis. Length of ERT also was seen to be inversely correlated with liver disease [5]. Our patient was not insured and could not afford ERT, although he did get it on compassionate grounds later which may have been too late to reverse the decompensated cirrhosis which had developed. Few cases of Gaucher disease needing liver transplantation for end-stage liver disease have been reported in the literature [3,6].

Ayto et al. in a case series of four patients reported excellent outcomes from liver transplantation for up to 10 years after the transplant with no evidence of Gaucher-related pathology in the graft. All patients had been on ERT and continued the same after transplant [6]. One of their patients needed a salvage TIPS for an uncontrolled, life-threatening variceal bleed. However, our case is the first Gaucher disease patient in literature to have received a TIPS for management of his refractory ascites. Liver transplantation can be performed in the setting of Gaucher disease for end-stage liver disease [6]. However, ERT needs to be continued lifelong to manage the other systemic effects of the disease as transplantation can only take care of the end-organ damage, i.e., the liver in this case. Our patient would have probably done well if he had received ERT earlier in the course of the disease and could have had the TIPS as a bridge to transplant with continuing ERT.

## Conclusions

Although hepatic manifestations such as hepatomegaly and hepatic fibrosis are very commonly seen, decompensated cirrhosis and portal hypertension leading to end-stage liver disease are not common in patients with Gaucher disease. Gaucher disease patients who have undergone splenectomy are more predisposed to developing liver-related complications. As established in our case, TIPS can be used to successfully treat refractory ascites in Gaucher-related decompensated liver disease as a bridge to liver transplantation with continuing ERT.
